# Microalgal glycerol-3-phosphate acyltransferase role in galactolipids and high-value storage lipid biosynthesis

**DOI:** 10.1093/plphys/kiad091

**Published:** 2023-02-21

**Authors:** Song Zou, Yuanchen Lu, Haiyan Ma, Yanhua Li, Guanqun Chen, Danxiang Han, Qiang Hu

**Affiliations:** Center for Microalgal Biotechnology and Biofuels, Institute of Hydrobiology, Chinese Academy of Sciences, Wuhan 430072, China; College of Advanced Agricultural Sciences, University of Chinese Academy of Sciences, Beijing 100049, China; Center for Microalgal Biotechnology and Biofuels, Institute of Hydrobiology, Chinese Academy of Sciences, Wuhan 430072, China; College of Advanced Agricultural Sciences, University of Chinese Academy of Sciences, Beijing 100049, China; Center for Microalgal Biotechnology and Biofuels, Institute of Hydrobiology, Chinese Academy of Sciences, Wuhan 430072, China; Center for Microalgal Biotechnology and Biofuels, Institute of Hydrobiology, Chinese Academy of Sciences, Wuhan 430072, China; Department of Agricultural, Food and Nutritional Science, University of Alberta, Edmonton AB T6G 2P5, Canada; Center for Microalgal Biotechnology and Biofuels, Institute of Hydrobiology, Chinese Academy of Sciences, Wuhan 430072, China; CAS Key Laboratory of Quantitative Engineering Biology, Shenzhen Institute of Synthetic Biology, Shenzhen Institute of Advanced Technology, Chinese Academy of Sciences, Shenzhen 518055, China; Faculty of Synthetic Biology, Shenzhen Institute of Advanced Technology, Chinese Academy of Sciences, Shenzhen 518055, China; Laboratory for Marine Biology and Biotechnology, Qingdao National Laboratory for Marine Science and Technology, Qingdao 266071, China

## Abstract

Glycerolipids are the most abundant lipids in microalgae, and glycerol-3-phosphate:acyl-CoA acyltransferase (GPAT) plays an important role in their biosynthesis. However, the biochemical and biological functions of algal GPAT remain poorly characterized. Here, we characterized the endoplasmic reticulum (ER)-associated GPAT of the model unicellular green alga *Chlamydomonas reinhardtii* (CrGPATer). Enzymatic assays indicated that CrGPATer is an *sn*-1 acyltransferase using a variety of acyl-CoAs as the acyl donor. Subcellular localization revealed that CrGPATer was associated with ER membranes and lipid droplets. We constructed overexpression (OE) and knockdown (KD) transgenic *C. reinhardtii* lines to investigate the in vivo function of CrGPATer. Lipidomic analysis indicated that CrGPATer OE enhanced the cellular content of galactolipids, especially monogalactosyldiacylglycerol, under nitrogen deficiency stress. Correspondingly, CrGPATer KD lines contained lower contents of galactolipids than the control. Feeding experiments with labeled phosphatidic acid revealed that the intermediate of the eukaryotic Kennedy pathway could be used for galactolipid biosynthesis in the chloroplasts. These results provided multiple lines of evidence that the eukaryotic Kennedy pathway mediated by CrGPATer may be involved in galactolipid biosynthesis in *C. reinhardtii*. OE of CrGPATer significantly increased the content of triacylglycerol and the yield of biomass. Moreover, the content and yield of 1, 3-olein-2-palmitin, a high-value lipid that can be used as an alternative for human milk fat in infant formula, were significantly enhanced in the OE transgenic lines. Taken together, this study provided insights into the biochemical and biological functions of CrGPATer and its potential as a genetic engineering target in functional lipid manufacturing.

## Teaser

The ER-associated glycerol-3-phosphate acyltransferase of *Chlamydomonas reinhardtii* is involved in the biosynthesis of galactolipids and functional triacylglycerol lipid 1, 3-olein-2-palmitin.

## Introduction

Microalgae have received extensive attention in recent years because of their ability to accumulate lipids and high-value products (Hu et al. 2008). The most abundant lipids in microalgae are glycerolipids, which are classified into membrane lipids (such as glycosylglycerides and phosphoglycerides) and storage lipids, including triacylglycerols (TAG), wax esters, and so on (Li-Beisson et al. 2016). Under favorable conditions, the contents of neutral lipids are very low in microalgal cells. While exposed to stress conditions (nitrogen deficiency, high light, etc.), many species of microalgae can accumulate TAG as an energy storage compound, accompanied by partial degradation of membrane lipids (Hu et al. 2008). The glycerolipids synthesis pathway is known as the Kennedy pathway. The de novo synthesized fatty acids in chloroplasts are sequentially esterified to the backbone of glycerol-3-phosphate (G-3-P) to form phosphatidic acid (PA), which is catalyzed by the G-3-P acyltransferase (GPAT) and lysophosphatidic acid acyltransferase (LPAAT). PA is a branch point of the glycerolipids synthesis pathway. On the one hand, PA is the precursor of anionic phosphoglycerides assembly, including phosphatidylglycerol and phosphatidylinositol. On the other hand, PA is dephosphorylated by phosphatidic acid phosphatase to form diacylglycerol (DAG), which is used for glycosylglycerides synthesis in the chloroplast, including monogalactosyldiacylglycerol (MGDG), digalactosyldiacylglycerol (DGDG), and sulfoquinovosyldiacylglycerol (SQDG), or for zwitterionic phosphoglycerides synthesis in the endoplasmic reticulum (ER), including phosphatidylcholine, phosphatidylethanolamine, phosphatidylserine, and betaine ether lipids. DAG can be also used to produce TAG, which is catalyzed by diacylglycerol acyltransferase (DGAT) (Chen et al. 2022). In addition, DAG can be converted to TAG via the acyl-CoA-independent pathway mediated by the phospholipid: diacylglycerol acyltransferase (PDAT) (for a recent review about algal lipid metabolism, see Li-Beisson et al. 2019).

As the first acyltransferase in the Kennedy pathway, GPAT exists in various organisms. According to its function, GPAT can be classified into 2 types that process the *sn*-1 or *sn*-2 esterification of G-3-P. Generally, GPATs involved in intracellular glycerolipids synthesis catalyze the *sn*-1 position esterification by using acyl-CoA or acyl-acyl carrier protein (acyl-ACP) as the acyl donors, thereby leading to the synthesis of *sn*-1 lysophosphatidic acid (LPA). This type of GPATs is ubiquitous in eukaryotic unicellular organisms, animals, and plants, and is located in various subcellular compartments, including ER (e.g. Sct1 and Gpt2 of yeast), mitochondria [e.g. human (*Homo sapiens*) GPAT1∼2], and chloroplasts [e.g. Arabidopsis (*Arabidopsis thaliana*) ATS1] (Wendel et al. 2009; Zhu et al. 2009; Kiegerl et al. 2019). GPATs that process the esterification of *sn*-2 are usually involved in the extracellular lipid synthesis, for which the main products are the *sn*-2 LPA, and the acyl donors are oxidized fatty acids (e.g. hydroxyl acyl-CoA and dicarboxylic acyl-CoA). This type of GPATs only exists in land plants and is associated with the ER (i.e. *A. thaliana* AtGPAT4∼8) and mitochondria (i.e. *A. thaliana* AtGPAT1∼3) for the biosynthesis of extracellular polyesters such as cutin and suberin (Jayawardhane et al. 2018). Due to the diversity of biological functions, GPAT has been considered as the potential genetic manipulation targets for biotechnological applications. Previous studies indicated that overexpression (OE) of the plastidial GPAT from cold-tolerant plants (e.g. *A. thaliana* ATS1) could increase the chill tolerance of cold-sensitive plants (Maraschin et al. 2019). OE of AtGPAT9 homolog could also improve the seed oil content of *A. thaliana* (Singer et al. 2016; Lv et al. 2020). In addition, heterologous expression of *Arxula adeninivorans* GPAT (SCT1) could maintain high levels of oleates in *Yarrowia lipolytica*, indicating the promising value of GPAT in modifying fatty acid profiles via biotechnology (Tsakraklides et al. 2018).

To date, all the identified microalgal GPATs are reported to locate in the chloroplasts and ER membranes, and most of them are involved in intracellular glycerolipids synthesis. However, their in vivo functions seem to be ambiguous. For example, under the nitrogen-depleted (N-depleted) conditions, the OE of the green microalga *Chromochloris zofingiensis* ER isoform *GPAT* (*CzGPAT2*) could promote TAG accumulation and, correspondingly, knockdown (KD) of this gene decreased the TAG content (Liu et al. 2019). However, altering the expression of *CzGPAT1*, a chloroplast isoform, does not affect the content of TAG in *C. zofingiensis* (Liu et al. 2019). Previous studies on the diatom *Phaeodactylum tricornutum* showed that the chloroplast-located PtGPAT1 promotes the accumulation of TAG under both the normal and stress conditions (Niu et al. 2016; Wang et al. 2018), and another predicted chloroplast isoform, PtGPAT2, could contribute to the TAG assembly under the normal conditions (Wang et al. 2020). The predicted PtGPAT3 ER isoform did not respond to the nitrogen deficiency induction (Wang et al. 2020). In addition, the chloroplast GPAT of the green microalga *Myrmecia incisa* (i.e. MiGPAT1) played a major role in membrane lipid synthesis (Ouyang et al. 2016), but the ER-located MiGPAT2 is mainly involved in TAG biosynthesis (Iskandarov et al. 2016; Sun et al. 2021).

As a model organism of microalgae, *Chlamydomonas reinhardtii* has been broadly used for lipid metabolism research (Merchant et al. 2007; Liu and Benning 2013a; Salome and Merchant 2019). Many key acyltransferases (i.e. CrDGAT2s, CrPDAT, CrLPAAT1, and CLPAAT2) of *C. reinhardtii* have been identified and characterized (Boyle et al. 2012; Yoon et al. 2012; Liu et al. 2016; Yamaoka et al. 2016; Kim et al. 2018), while the CrGPATs remained largely uncovered. There are only 2 predicted *GPAT* in the *C. reinhardtii* genome, namely *CrGPATcp* and *CrGPATer*, respectively. The 2 genes were cloned from the *C. reinhardtii* cDNA library and their GPAT activity was preliminarily confirmed with in vitro assay (Duarte-Coello et al. 2019). CrGPATcp that contains a typical chloroplast transit peptide is the homolog of the *A. thaliana* chloroplast-located soluble GPAT (ATS1). CrGPATer is homologous to AtGPAT9, which was associated with ER and participated in the intracellular glycerolipid synthesis in *A. thaliana* (Singer et al. 2016). In addition, CrGPATer was identified in the proteome of the lipid droplets of *C. reinhardtii* (Nguyen et al. 2011; Goold et al. 2015). However, comprehensive investigation on the functions of CrGPAT is still lacking, which hinders its biotechnical applications in microalgae.

In this study, enzyme activities and substrate preference were analyzed and the results revealed that CrGPATer prefers acyl-CoA as substrates and produces *sn*-1 LPA, a key intermediate lipid in glycerolipid biosynthesis in the Kennedy pathway. Subcellular localization analysis found that, unlike the predicted ER location, CrGPATer is a dual-targeting protein localized at both ER and lipid bodies. Subsequently, CrGPATer OE and KD *C. reinhardtii* lines were generated and used in lipidomic analysis and other analyses to comprehensively investigate the in vivo functions of CrGPATer. The results indicated that CrGPATer was essential for both intracellular TAG and galactolipids synthesis in *C. reinhardtii*. In terms of the potential application of CrGPATer in algal lipid biotechnology, the results revealed that *CrGPATer* OE could significantly enhance the content and yield of 1, 3-olein-2-palmitin (OPO), an essential component in human milk fat, in *C. reinhardtii*.

## Results

### CrGPATer is an *sn*-1 acyltransferase preferring acyl-CoA as the substrate

**Figure 1.**
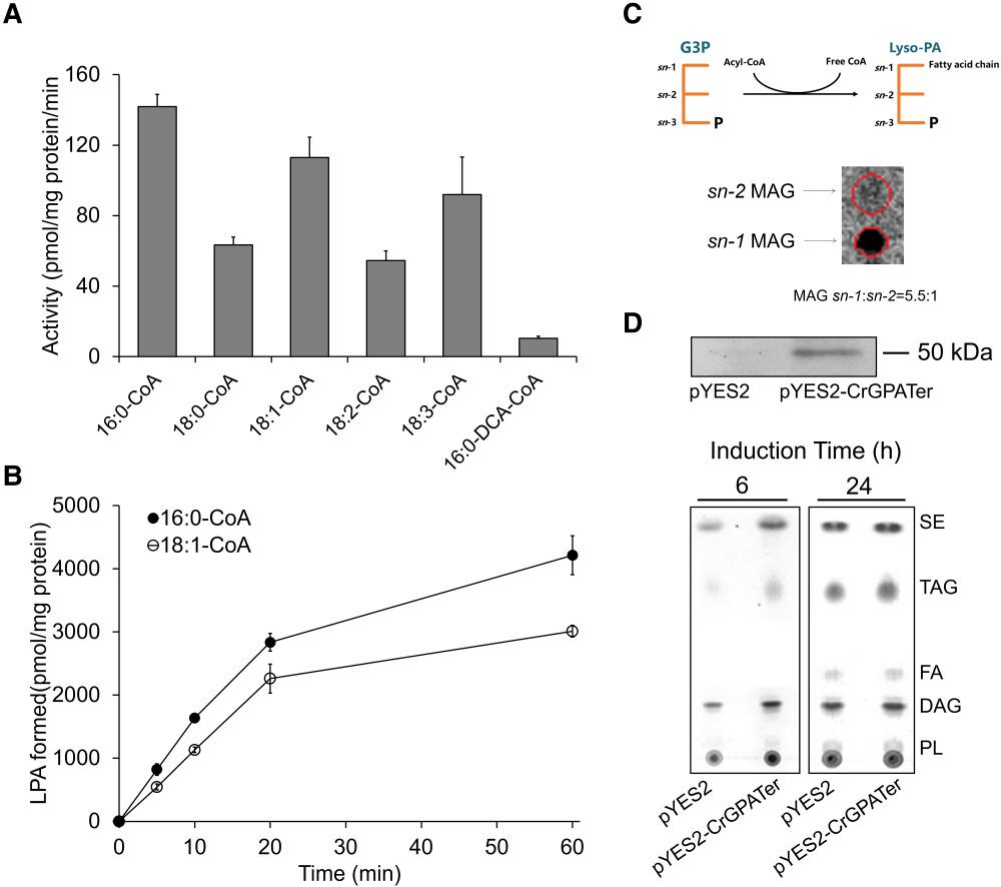
In vitro and ex vivo activities of CrGPATer. **A)** In vitro enzyme assays of CrGPATer and its substrate preference to various acyl-CoAs and 16:0-DCA-CoA. The bar value represents mean enzyme activities assayed at 22 °C for 20 min. The error bar of 3 independent enzyme preparations is also shown. **B)** CrGPATer prefers 16:0-CoA (solid circle) than 18:1-CoA (empty circle) as a substrate to form LPA in vitro. The mean enzyme activities were assayed at 22 °C and standard errors are based on 3 independent enzyme preparations. **C)** In vitro regiospecificity assays of CrGPATer. **D)** The major polar lipid classes TLC analysis in CrGPATer-overexpressed *Saccharomyces cerevisiae Δgat1*. Whole protein was resolved in SDS-PAGE followed by Western analysis with anti-His antibody and TLC analysis of total lipids extracted from transformant with the overexpressed CrGPATer and control. G3P, glycerol-3-phosphate; PA, phosphatidic acid t; *P*, phosphate group. SE, sterol esters; TAG, triacylglycerol; FA, fatty acids; DAG, diacylglycerol; PL, phospholipids.

CrGPATer is a putative ortholog of AtGPAT9 which has been reported to possess the *sn*-1 acyltransferase activity to incorporate acyl-CoAs into glycerol-3-phosphate in the Kennedy pathway (Singer et al. 2016). The recombinant CrGPATer was expressed in the *Saccharomyces cerevisiae Δgat1* mutant and the crude membranes containing the recombinant protein were extracted from the yeast cells harvested at 18 h for enzymatic assay (Zheng et al. 2003; Yang et al. 2010; Singer et al. 2016). The enzymatic activity was 141.8, 63.4, 113.1, 54.6, and 91.9 pmol LPA·mg protein^−1^·min^−1^ (corrected in each case for residual background activity in empty vector controls), respectively, with 16:0-CoA, 18:0-CoA, 18:1-CoA, 18:2-CoA, and 18:3-CoA, as the substrate, indicating that 16:0-CoA was the most favorite substrate of CrGPATer, followed by 18:1-CoA (Fig. 1A), which is further confirmed in the time course experiment (Fig. 1B). Notably, GPAT activity was very low (10.4 pmol LPA·mg protein^−1^· min^−1^) when 16:0-dicarboxylic acid (DCA)-CoA (the precursor of cutin/suberin) was added as the acyl donor, indicating that CrGPATer was not involved in polyesters biosynthesis. Since the GPAT in the Kennedy pathway for glycolipid biosynthesis should specifically add the acyl-chain to the *sn-*1 position of G-3-P, we further analyzed the regiospecificity of the CrGPATer. As shown in Fig. 1C, when the produced LPA was dephosphorylated and converted to stable MAG and then separated on borate-thin layer chromatography (TLC) plate, the majority of MAG was *sn*-1 MAG (*sn-*1 MAG vs *sn-2* MAG = 5.5:1). Therefore, the enzymatic assay results indicated that CrGPATer was an *sn*-1 acyltransferase preferring 16:0-CoA as the substrate, which may contribute to glycerolipid biosynthesis.

### OE of *CrGPATer* in *S. cerevisiae Δgat1* enhanced the production of TAG

To confirm the ex vivo function of *CrGPAT*er involved in glycerolipid biosynthesis, the recombinant His-tagged CrGPATer was overexpressed in *S. cerevisiae Δgat1*. Immuno-blotting with anti-His antibodies revealed an overexpressed protein with an apparent molecular mass of ca. 50 kDa, which was consistent with the predicted molecular mass of CrGPATer (ca. 51.3 kDa) (Fig. 1D). A TLC analysis of total lipids confirmed that the TAG and sterol esters content was enhanced in *S. cerevisiae Δgat1* with the OE of CrGPATer when compared with the control (transformed with an empty vector) in the early exponential phase (6 h) (Fig. 1D). However, there was no substantial difference in TAG and sterol esters observed after 24 h of induction (Fig. 1D). These results indicated the functions of CrGPATer OE in TAG and sterol esters accumulation could be better observed during the early exponential phase of *S. cerevisiae Δgat1*.

### CrGPATer is localized at ER and lipid body

**Figure 2.**
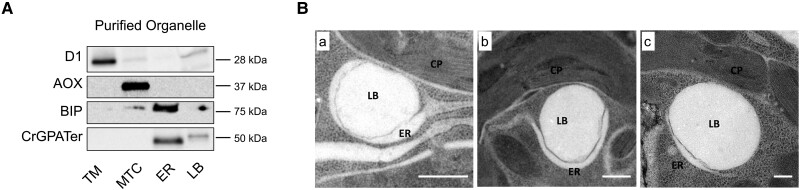
CrGPATer is a dual-targeting protein localized at ER and lipid body. **A)** Subcellular localization of CrGPATer. cc400 cells grown under nitrogen-depleted conditions for 1 d were used for organelle preparation. TM, thylakoid membrane; MTC, mitochondria; LB, lipid body. D1, AOX, and BIP were used as markers for the TM, MTC, and ER, respectively. **B)** During the nitrogen-depleted condition, ER contact with LB based on transmission electron micrographs. Bars = 1 μm. LB, lipid body; CP, chloroplast.

The subcellular localization prediction results obtained by using SignalP and TargetP showed that no transit peptide or signal peptide was found in the CrGPATer sequence. While ProtComp generated a high score (9.0/10.0) that indicated CrGPATer was located at ER. To examine the subcellular localization of CrGPATer experimentally, we prepared subcellular fractions of *C. reinhardtii* cc400, including thylakoid membranes, mitochondria, ER, and lipid droplets. Firstly, the marker proteins for each subcellular compartment were found to be enriched in the corresponding purified organelles (Fig. 2A). By using the polyclonal antibody of CrGPATer, the target protein was detected in both the ER and lipid droplets fractions (Fig. 2A). Though the delipidation was performed by using acetone, the lipids cannot be completely removed from the sample, which caused the changed mobility of lipid droplets-associated CrGPAT on the SDS-PAGE. Furthermore, transmission electron microscopic observations showed that there was a connection between lipid droplets and ER under N-depleted conditions (Fig. 2B).

### TAG accumulation in the transgenic *C. reinhardtii* cc400 with OE or KD of CrGPATer

**Figure 3.**
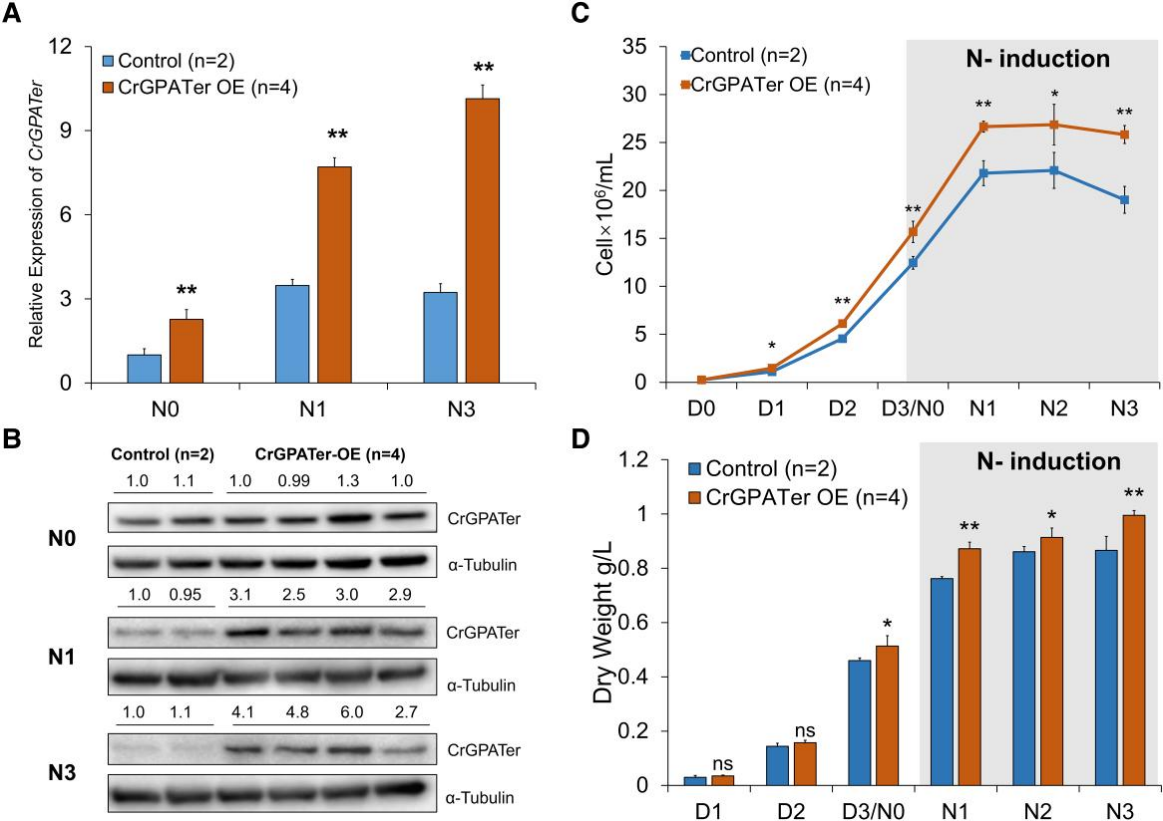
OE of CrGPATer in *C.reinhardtii* strain cc400. Relative mRNA expression level of *CrGPATer***A)** and its protein abundance **B)** in the control group and CrGPATer OE lines during nitrogen-replete (N0) and nitrogen-depleted (N1, N3) conditions. RT-qPCR data normalized to target gene expression in the control group at N0. Western blot data normalized to CrGPATer expression in the control group at each time point and the relative gray value was analyzed by Image Lab software (Bio-Rad, USA). α-Tubulin was used as a reference in both RT-qPCR and WB assays. **C)** Cell number of the control group and CrGPATer OE lines in TAP medium. Culture started with 2.5 × 10^5^ cells·mL^−1^ in normal TAP medium (D1, D2, and D3/N0) and N depleted after 3 d (N1, N2, and N3). Control (*n* = 2) contains wild-type cc400 and one empty vector transformant. CrGPATer OE (*n* = 4) contains 4 individual transformants. Every kind of strain had 2 biological parallels in the culture cycle. Data are means of replicates with Sd. Asterisks indicate statistically significant differences from the control group based on Student's *t*-test (ns: *P* > 0.05; *P < 0.05, **P < 0.01).

**Figure 4.**
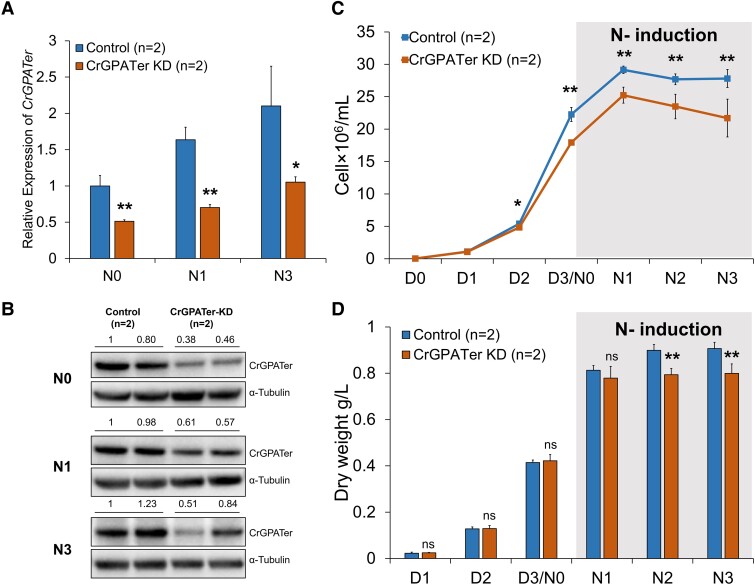
KD of CrGPATer in *C.reinhardtii* strain cc400. Relative mRNA expression level of CrGPATer **A)** and its protein abundance **B)** among control group and CrGPATer KD lines during nitrogen-replete (N0) and nitrogen-depleted (N1, N3) conditions. RT-qPCR data normalized to target gene expression in the control group at N0. WB data normalized to CrGPATer expression in the control group at each time point and the relative gray value was analyzed by Image Lab software (Bio-Rad, USA). α-Tubulin was used as a reference in both RT-qPCR and WB assays. Cell number **C)** and dry weight **D)** of the control group and CrGPATer KD lines in normal TAP medium (D1, D2, and D3/N0) and N depleted after 3 d (N1, N2, and N3). Control (*n* = 2) contains wild-type cc400 and one empty vector transformant. CrGPATer KD (*n* = 2) contains 2 individual transformants. Every kind of strain had 2 biological parallels in the culture cycle. Data are means of replicates with Sd. Asterisks indicate statistically significant differences from the control group based on Student's *t*-test (ns: *P* > 0.05; *P < 0.05, **P < 0.01).

In order to characterize the function of *CrGPAT*er in vivo, CrGPATer OE and KD lines of *C. reinhardtii* cc400 strain were generated. According to the reverse transcription quantitative real-time PCR (RT-qPCR) and Western blotting results, there were 2 individual KD transformants and 4 individual OE transformants screened from the mutants with fully inserted recombinant fragments. Compared to the control (wild type and an empty vector transformant), the expression of CrGPATer in the OE lines increased by 1.3 to 2.1 times, while the expression of CrGPATer in the KD lines showed 35.7% to 57.1% reduction under N-replete condition (N0) and N-depleted conditions (N1 and N3) (Figs. 3, A and B and Fig. 4, A and B).

The algal cells were cultured in nitrogen-replete Tris-acetate-phosphate (TAP) growth medium for 3 d, followed by 3 d under the N-depleted conditions. As shown in Fig. 3, C and D, the cell growth of the OE lines was faster than that of the control group. The cell density of OE lines reached 1.47, 6.11, and 15.6 × 10^6^ cells mL^−1^ on Days 1, 2, and 3, respectively, under N-replete conditions and significantly higher (*P* < 0.05) than that of control in each time point (Fig. 3C). Under nitrogen deficiency stress, the OE lines still has higher cell density and dry weight. The cell density of OE lines reached 26.6, 26.8, and 25.8 × 10^6^ cells mL^−1^ and the dry weight reached 0.87, 0.91, and 0.99 g·L^−1^ on Days 1, 2, and 3, respectively, under N-depleted conditions which were significantly higher (*P* < 0.05) than that of control in each time point (Fig. 3, C and D). On the other hand, knocking down the CrGPATer expression impaired the cell growth (Fig. 4, C and D). Before nitrogen deficiency, the cell density was ca. 17.9 × 10^6^ cells mL^−1^, which was 80.4% that of the control group. And during the nitrogen deficiency induction, the growth of KD lines was continuously suppressed, which only reached 86%, 85%, and 78% of that of the control group over 3 d under N-depleted conditions (Fig. 4C). As for the biomass accumulation, the dry weight of the KD lines was slightly reduced when compared with that of the control, which was decreased by 12.6% and 13.4% on Days 2 and 3 after being subject to the N-depleted conditions, respectively (Fig. 4D).

**Figure 5.**
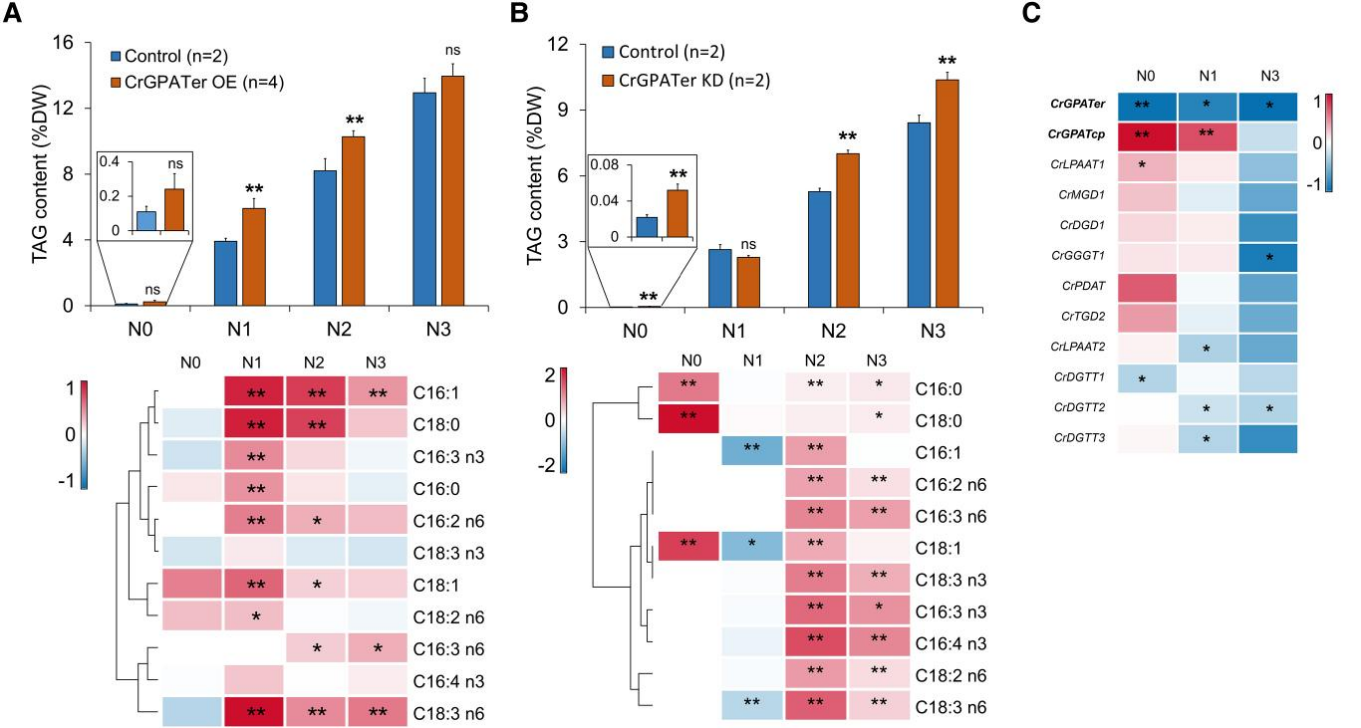
TAG accumulation in CrGPATer OE and KD transgenic lines. TAG content (%, per dry weight) of the control group and CrGPATer OE lines **A)** or CrGPATer KD lines **B)** during nitrogen-replete (N0) and nitrogen-depleted (N1, N2, and N3) conditions, measured by GCMS. The heatmap indicates the fold change, Log_2_(Content _OE or KD_/Content _Control_), of fatty acids content of TAG. **C)** Several glycerolipid metabolism-related gene expression (relative mRNA level) of CrGPATer KD lines compared with the control group. The heatmap indicates the fold change, Log_2_(KD/Control), of relative gene expression. Control (*n* = 2), CrGPATer OE (*n* = 4), and CrGPATer KD (*n* = 2) are described in Figs. 3 and 4. Data are means of replicates with Sd. Asterisks indicate statistically significant differences from the control group based on Student's *t*-test (ns: *P* > 0.05; *P < 0.05, **P < 0.01).

Compared to the control group, the TAG content (% per dry weight) in the CrGPATer OE lines increased by 50.7% and 25.3% on Day 1 and Day 2 under N-depleted conditions, respectively, but was not significantly changed under N-replete conditions and on Day 3 under N-depleted conditions (Fig. 5A). Similarly, the contents of various TAG-associated fatty acids showed significant increases on Days 1, 2, and 3 under N-depleted conditions, but were comparable with that of the control under N-replete conditions. Eight fatty acids exhibited higher contents than that of the control on Day 1 under N-depleted conditions, among which C18:3 n6 was the species with the greatest increase, followed by C18:0 and C16:1, which was 2.2, 1.9, and 1.9 times (*P* < 0.05) of that of the control. However, when the nitrogen deficiency induction was sustained, 6 species of fatty acids showed significant increases on Day 2, which were C16:1, C16:2 *n*6, C18:0, C18:1, and C18:3 *n*6, and only 3 species of fatty acids including C16:1, C16:3 *n*6, and C18:3 *n*6 showed significant increases on Day 3 (Fig. 5A).

Unexpectedly, under the N-replete conditions, the TAG content of KD lines increased by 2.4 times when compared with that of the control (*P* < 0.05). During nitrogen deficiency induction, there was no significant alteration of TAG content between KD lines and the control group on Day 1 under N-depleted conditions, while significant increases (*P* < 0.05) in the TAG contents were observed in the KD lines on Day 2 and Day 3 under N-depleted conditions (increased by 32.7% and 23.2%, respectively) (Fig. 5B). In terms of the fatty acid profiles of TAG, when compared with the control group, the saturated (C16:0 and C18:0) and monounsaturated species (C18:1) significantly increased (*P* < 0.01) by 1.2, 3.1, and 2.1 times under N-replete conditions, respectively. On Day 1 under N-depleted conditions, the content of C16:1, C18:1, and C18:3 n6 that are associated with TAG decreased significantly which was 45.2% (*P* < 0.01), 58.9% (*P* < 0.05), and 67.5% (*P* < 0.01) of that of control and the content of other fatty acids has no significant alteration compared with the control group. Subsequentially, the content of most fatty acid species associated with TAG significantly increased on Day 2 under N-depleted conditions except for C18:0. Among these fatty acids, C16:4 *n*3 was the species with the greatest increase, followed by C18:3 *n*6 and C16:3 *n*3, which was 2.8 (*P* < 0.01), 2.5 (*P* < 0.01), and 2.3 (*P* < 0.01) times of that of the control. On Day 3 under N-depleted conditions, except for C16:1 and C18:1, the content of the other TAG-associated fatty acids also was significantly higher than that of control, among which C16:4 *n*3 still was the species with the greatest increase, followed by C18:3 *n*6 and C16:3 *n*3, which was 2.0 (*P* < 0.01), 1.8 (*P* < 0.05), and 1.7 (*P* < 0.01) times of that of the control, respectively (Fig. 5B).

The expression of several key genes related to the glycerolipid metabolism in the CrGPATer KD lines was determined by using RT-qPCR. The results showed that the expression of *CrGPATcp* encoding an isoenzyme of CrGPATer in the prokaryotic Kennedy pathway was significantly upregulated by 2.2 and 1.6 times in the CrGPATer KD lines under N-replete and N-depleted conditions, respectively, but the expression of the other genes involved in lipid metabolism was not altered (Fig. 5C). The increased TAG contents of CrGPATer KD lines might result from the enhanced expression of prokaryotic Kennedy pathway.

### CrGPATer is involved in the synthesis of galactolipids

**Figure 6.**
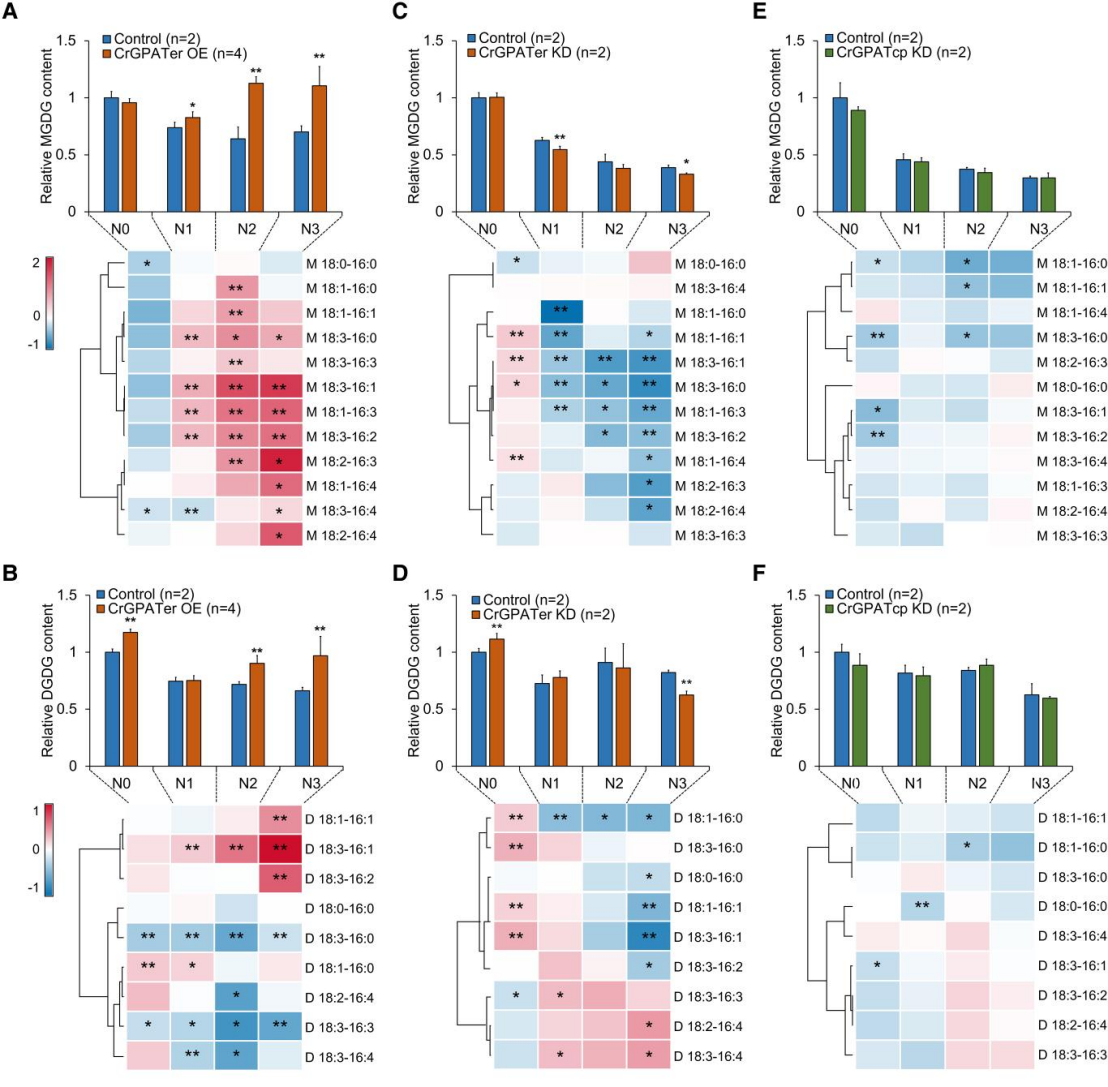
Galactolipids accumulation in CrGPATer OE, CrGPATer KD, and CrGPATcp KD transgenic lines. The fold change of galactolipids among CrGPATer OE **(A** and **B)**, CrGPATer KD (**C** and **D)**, and CrGPATcp KD **(E** and **F)** transgenic lines, compared with the control group during nitrogen-replete (N0) and nitrogen-depleted (N1, N2, and N3) conditions. Histograms indicate the relative changes in the galactolipid contents of transgenic lines and the control group under N-depleted conditions when compared with that of the control group under N-replete conditions. Data are means of replicates with Sd. Control (*n* = 2), CrGPATer OE (*n* = 4), and CrGPATer KD (*n* = 2) are described in Figs. 3 and 4. CrGPATcp KD (*n* = 2) is described in Supplemental Fig. S1. The heatmaps indicate compared with the control group, the fold change, Log_2_(Content _transgenic line_/Content _Control_), of each compound contents in 3 transgenic lines. Compound species of galactolipids are written as “M or D *sn*-1 FA-*sn*-2 FA.” M means MGDG and D means DGDG. Data are means of replicates and asterisks indicate statistically significant differences from the control group based on Student's *t*-test (*P < 0.05, **P < 0.01).

The galactolipids, including MGDG and DGDG, are the main components of thylakoid membranes. The contents of these 2 galactolipids in CrGPATer OE and KD lines were determined by using ultraperformance liquid chromatography-tandem mass spectrometry (UPLC-MS/MS). As shown in Fig. 6A, under normal culture conditions, the contents of MGDG in the CrGPATer OE lines were not significantly different from that in the control group but were significantly higher than that in the control group, which reached ca. 1.1, 1.8, and 1.6 times of that of the control group on Days 1, 2, and 3 under the N-depleted conditions, respectively (Fig. 6A). The contents of 12 MGDG species have been tracked both in CrGPATer OE lines and control group. As shown in the heatmap, MGDG 34:3 (18:3/16:0), MGDG 34:4 (18:3/16:1, 18:1/16:3), and MGDG 34:5 (18:3/16:2) increased significantly on Day 1 under the N-depleted conditions. With the continuation of nitrogen deficiency induction, except for MGDG 34:0 (18:0–16:0), MGDG 34:5 (18:1/16:4), MGDG 34:6 (18:2/16:4), and MGDG 34:7 (18:3/16:4), the contents of most MGDG species increased significantly on Day 2 under N-depleted conditions. Among these species, MGDG 34:4 (18:1/16:3) showed the highest increase, 1.2-fold higher than the control group. On Day 3 under the N-depleted conditions, the contents of the species including MGDG 34:5 (18:1/16:4), MGDG 34:6 (18:2/16:4), and MGDG 34:7 (18:3/16:4) increased significantly when compared with that of the control, which was not elevated before Day 3 (Fig. 6A).

As for the DGDG content, significant increases (*P* < 0.05) in CrGPATer OE lines were observed both under both the N-replete and N-depleted conditions. Compared with that of the control group, the DGDG content of OE lines increased by 17.3% under N-replete conditions and by 25.7% and 46.4% on Days 2 and 3 under N-depleted conditions, respectively (Fig. 6B). Among the 9 DGDG species, DGDG 34:4 (18:3/16:1) was the only species of which the content continuously increased significantly under the N-depleted conditions, and its content was higher than that of the control by 16.1%, 48.7%, and 143% on Days 1, 2, and 3, respectively. Besides, on Day 3 under N-depleted conditions, significant increases (*P* < 0.05) were observed in DGDG 34:2 (18:1/16:1) and DGDG 34:5 (18:3/16:2), which increased by 38.2% and 59.4%, respectively, when compared with that of the control. In contrast, the contents of DGDG 34:3 (18:3/16:0) and DGDG 34:6 (18:3/16:3) reduced under both the N-replete and N-depleted conditions. Significant decreases (*P* < 0.05) were observed in DGDG 34:6 (18:2/16:4) on Day 2 and DGDG 34:7 (18:3/16:4) on Days 2 and 3 under N-depleted conditions (Fig. 6B).

In contrast to the increases in the contents of most galactolipid species in the CrGPATer OE lines, the galactolipids contents significantly decreased in the CrGPATer KD lines when compared with that of the control under the N-depleted conditions (Fig. 6, C and D). For MGDG, significant decreases (*P* < 0.05) in the contents were observed on Day 1 and Day 3 under the N-depleted conditions (decreased by 12.8% and 15.1%, respectively) (Fig. 6C). For DGDG, the content was 11.4% higher in the KD lines than that of the control group under the N-replete conditions, but reduced by 23.9% on Day 3 under N-depleted conditions (Fig. 6D). The fold-changes in the content of 12 MGDG species and 9 DGDG species in the KD line when compared with that of the control are shown in Fig. 6, C and D heatmaps. Though significant increases (*P* < 0.05) were observed in MGDG 34:2 (18:1/16:1), MGDG 34:3 (18:3/16:0), MGDG 34:4 (18:3/16:1), and MGDG 34:5 (18:1/16:4) under the N-replete conditions, none of the MGDG species in the CrGPATer KD lines exhibited the elevated contents when the CrGPATer KD lines were subjected to N-depletion. On Day 1 under the N-depleted conditions, the content of MGDG 34:1 (18:1/16:0), MGDG 34:2 (18:1/16:1), MGDG 34:3 (18:3/16:0), and MGDG 34:4 (18:3/16:1, 18:1/16:3) reduced significantly. On Day 2, the content of MGDG 34:3 (18:3/16:0) and MGDG 34:4 (18:3/16:1, 18:1/16:3) also showed significant reduction and MGDG 34:5 (18:3/16:2) declined by 27.9% when compared with that of the control. On Day 3, the content of most MGDG species showed significant reduction which were MGDG 34:2 (18:1/16:1), MGDG 34:3 (18:3/16:0), MGDG 34:4 (18:3/16:1, 18:1/16:3), MGDG 34:5 (18:1/16:4, 18:2/16:3, 18:3/16:2), and MGDG 34:6 (18:2/16:4) (Fig. 6C).

For DGDG, the content of DGDG 34:1 (18:1/16:0), DGDG 34:2 (18:1/16:1), DGDG 34:3 (18:3/16:0), and DGDG 34:4 (18:3/16:1) in the CrGPATer KD lines increased by 16.3%, 13.9%, 25.8%, and 26.2%, respectively, under the N-replete conditions. After nitrogen deficiency, when compared with that of the control, the content of DGDG 34:1(18:1/16:0) decreased by 27.1%, 29.1%, and 30.1% (*P* < 0.05) on Days 1, 2, and 3 upon nitrogen deficiency, respectively. Besides, on Day 3, significant decreases (*P* < 0.05) were observed in the content of DGDG 34:0 (18:0/16:0) DGDG 34:2 (18:1/16:1), DGDG 34:4 (18:3/16:1), and DGDG 34:5 (18:3/16:2), which was reduced by 15.6%, 29.9%, 43.9%, and 22.3% than that of the control, respectively. In contrast, for the DGDG species with high degrees of desaturation, such as 34:6 (18:3/16:3, 18:2/16:4) and 34:7 (18:3/16:4), their contents were higher than the control group under the N-depleted conditions (Fig. 6D).

CrGPATer was proved to be an acyltransferase in the eukaryotic Kennedy pathway and located in the ER and lipid body (Figs. 1 and 2). And the above results suggested that CrGPATer might be involved in the biosynthesis of galactolipids. However, galactolipids were generally considered to be synthesized from PA produced from the prokaryotic Kennedy pathway in the chloroplast (Liu and Benning 2013b). Thus, CrGPATcp KD lines were introduced to investigate the role of CrGPATcp in galactolipids production (Supplemental Fig. S1). The results showed that the KD of CrGPATcp did not significantly alter the content of either MGDG or DGDG under both N-replete and N-depleted conditions (Fig. 6, E and F). In terms of the specific galactolipids species, the contents of most MGDG and DGDG molecules were not significantly altered under N-replete and N-depleted conditions (Fig. 6, E and F). Taken together, these data indicated that the CrGPATer was involved in the synthesis of galactolipids in *C. reinhardtii*.

### *C. reinhardtii* can utilize extraplastidial PA for galactolipids synthesis

In chloroplast, DAG was galactosylated by MGDG synthase (MGD) to form MGDG and MGDG could be galactosylated again to form DGDG by DGDG synthase (DGD). And previous study proved that both the CrMGD and CrDGD were located in the chloroplast membrane (Warakanont et al. 2015). Both prokaryotic and eukaryotic Kennedy pathways could provide PA to form DAG, but the origin of PA used for galactolipids synthesis is unclear yet.

**Figure 7.**
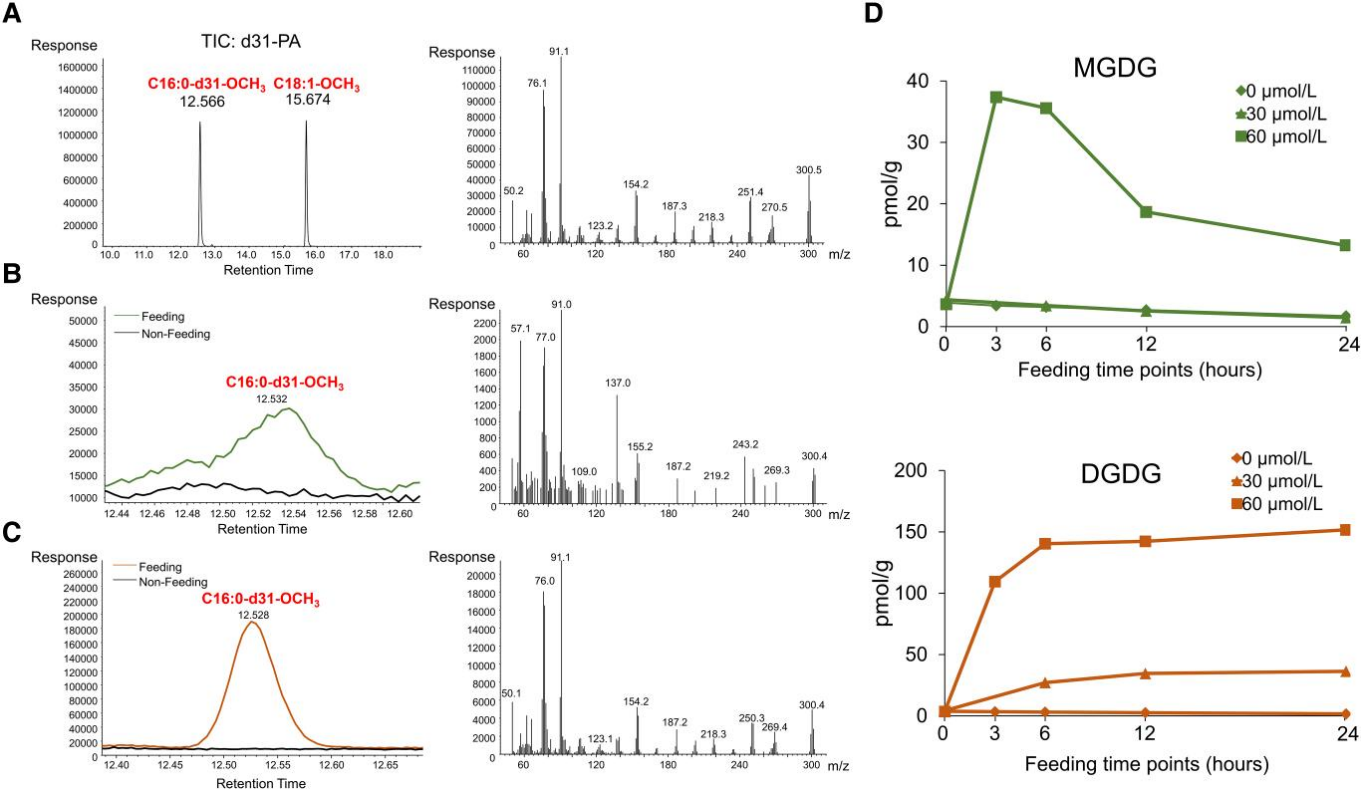
*Chlamydomonas reinhardtii* utilizes extra-chloroplastic PA for MGDG and DGDG biosynthesis. **A)** Analysis of 16:0-d31-18:1 PA by GCMS. Two fatty acid methyl ester (FAME) products of d31-PA are shown in the gas chromatogram (left panel) and the fragment peaks of d31-C16:0-OCH_3_ are represented in the mass chromatogram (right panel). After feeding 60 *μ*mol·L^−1^ d31-PA, the unique methylated product, d31-C16:0-OCH_3_ can be detected in the MGDG of the 3-h time point sample **B)** and the DGDG of the 24-h time point sample **C)**, and the blank line and green (MGDG) or orange (DGDG) line represent nonfeeding and feeding sample. The mass spectrum of C16:0-d31-OCH_3_ is shown on the right panel. **D)** Content of labeled MGDG and DGDG after feeding 24 h with 0, 30, and 60 μmol·L^−1^ d31-PA.

Given that in vivo functional analysis indicated that CrGPATer was responsible for the galactolipids, the *C. reinhardtii* cells were fed with the deuterated PA (d31-PA) that contains one d31-C16:0 and one normal C18:1 fatty acid chain at the *sn*-1 and *sn*-2 position, respectively, with the aim to verify whether the extraplastidial PA for galactolipid biosynthesis was occurring in the chloroplasts. In terms of the methodology establishment, the methyl esterification product of d31-PA was qualitatively measured by using GC/MS first. The retention time of d31-C16:0 methyl esterified product was at *ca*. 12.57 min, and the mass spectrum on the right panel showed the species and abundance of its specific fragment ions (Fig. 7A). After feeding 60 *μ*mol·L^−1^ d31-PA, the galactolipids were separated from the extracted total lipids by using TLC and measured with GC/MS. The methyl esterification products of d31-C16:0 were detected in the fractions of MGDG and DGDG after feeding the algal cells with d31-PA for 3 and 24 h, respectively, and the corresponding mass spectrum of ion product were consistent with that of the standard (Fig. 7, B and C). The results of the quantitative analysis showed that the incorporation of d13-PA into galactolipids was concentration-dependent. The content of the labeled MGDG and DGDG was higher when feeding with 60 *μ*mol·L^−1^ than that of 30 *μ*mol·L^−1^, respectively (Fig. 7D). The content of the labeled MGDG reached the maximum of ca.40 pmol·g^−1^ dry weight at 3 h after feeding, and then decreased to 13 pmol·g^−1^ dry weight at 24 h. In contrast, the content of the labeled DGDG progressively increased to *ca*. 140 pmol·g^−1^ dry weight at 6 h after feeding and then stabilized at this level until 24 h. In addition, the content of the labeled DGDG was 3 to 10 times higher than that of the labeled MGDG from 3 to 24 h. In summary, all these results indicated that *C. reinhardtii* was able to utilize the extraplastidial PAs to synthesize the galactolipids within the chloroplast.

### OE of CrGPATer enhanced the production of OPO in *C. reinhardtii*

**Figure 8.**
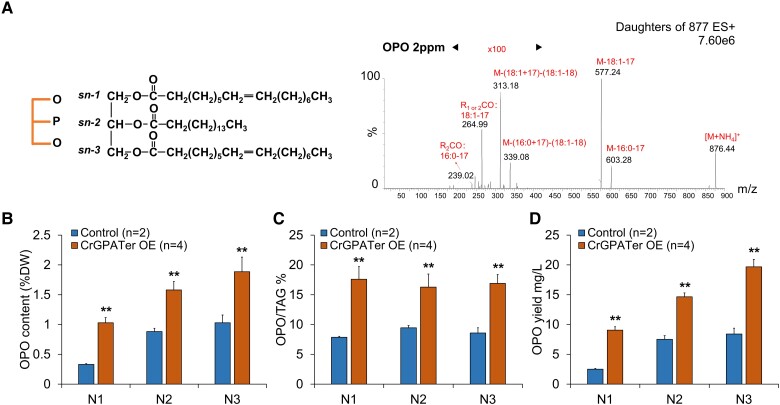
OE of CrGPATer increases the content of OPO in both dry weight and TAG pool. **A)** OPO chemical structure and the product ion spectrum of the [M + NH_4_]^+^ ion at m/z 876.4. OPO content (% per dry weight) **B)**, its proportion of total TAG **C)**, and the yield of OPO **D)** in CrGPATer OE transgenic lines and control group during nitrogen-depleted (N1, N2, and N3) conditions. Control (*n* = 2) and CrGPATer OE (*n* = 4) are described in Fig. 3. Data are means of replicates with Sd. Asterisks indicate statistically significant differences from the control group based on Student's *t*-test (ns: *P* > 0.05, *P < 0.05, **P < 0.01).

OPO is a functional lipid in human milk which plays an important role in the healthy growth and development of infants, and *C. reinhardtii* could synthesize OPO naturally based on our previous research (Lu et al. 2022). To quantitatively analyze the content of OPO, the multiple reaction monitoring (MRM) mass spectrometry method was established. The product-ion spectrum of the ammonium adducts ion of OPO (i.e. [M + NH_4_]^+^, *m*/*z* 876) is shown in Fig. 8A. The ion of *m*/*z* 313 showed the highest abundance among the products, which corresponded to the *sn*-2 16:0 MAG formed due to the loss of 2 fatty acyl groups, and thus it was selected for the scanning analysis of OPO. To test the potential functions of CrGPATer in OPO production, the OPO contents were analyzed in the negative contrail and CrGPATer OE lines. As shown in Fig. 8B, the maximum content of OPO in the CrGPATer OE lines reached ca. 1.88% of the dry weight at Day 3 under the N-depleted conditions, which is 80% higher than that of the control (*P* < 0.01). The portion of OPO in TAG was ca. 17% under the N-depleted conditions in the CrGPATer OE lines, which was nearly 1-fold higher than that of the control (*P* < 0.01) (Fig. 8C). In addition, the biomass accumulation of the CrGPATer OE lines was 0.99 g·L^−1^ at Day 3 under N-depleted conditions, which was 15% more than that of the control group (*P* < 0.01) (Fig. 3D). Taken together, the OPO yield of CrGPATer OE lines reached 9.06, 14.65, and 19.67 mg·L^−1^ at Days 1, 2, and 3 under the N-depleted conditions, respectively, which was 3.6, 1.9, and 2.3 times that of the corresponding control group (*P* < 0.01) (Fig. 8D). These data suggested that overexpressing CrGPATer enhanced the OPO production in *C. reinhardtii*.

## Discussion

### The relationship between TAG synthesis and the Kennedy pathway in *C. reinhardtii*

In plants and non-photosynthetic cells such as yeast and mammalian cells, the final assembly of TAG is found to be accomplished in the ER (Wakimoto et al. 2003; Shockey et al. 2006; Cao et al. 2007). As the precursor of esterification reaction in the last step of TAG synthesis, there is no doubt that the DAG pool can be provided by both prokaryotic and eukaryotic Kennedy pathways (Li-Beisson et al. 2015), but it is unclear about the respective contribution of these 2 pathways to TAG biosynthesis in *C. reinhardtii*. A previous study indicated that TAG biosynthesis is largely dependent on the chloroplast pathway in *C. reinhardtii* mainly based on the fatty acyl groups at the *sn*-2 position of the glycerol backbone (Fan et al. 2011). However, this study demonstrated the eukaryotic Kennedy pathway was responsible for the bulk of TAG biosynthesis in the microalga *C. reinhardtii*.

Firstly, CrGPATer is distinguished from land plant GPATs (e.g. *A. thaliana* AtGPAT1∼8) which are found to be involved in the extracellular lipids (i.e. polyesters) synthesis (Jayawardhane et al. 2018). CrGPATer has strong selectivity for acyl-CoA donor other than the DCA-acyl-CoA (Fig. 1, A and B) and when CrGPATer was overexpressed in *S. cerevisiae Δgat1* mutant, the content of TAG and sterol esters was higher than that of control (Fig. 1D). These results indicated that CrGPATer was responsible for the intracellular lipids biosynthesis. Furthermore, OE of the eukaryotic type CrGPATer led to the enhanced TAG biosynthesis (Fig. 5A), which was consistent with the results from genetic manipulation of its homologous genes in other organisms (Singer et al. 2016; Fukuda et al. 2018). Moreover, the contents of most TAG-associated fatty acids increased, indicating that CrGPATer OE can enhance the carbon flow into the Kennedy pathway. It was found that CrGPATer is present in both the lipid droplets and the ER membranes (Fig. 2A), and these 2 organelles are connected with each other as indicated by the ultrastructure observation (Fig. 2B). This observation was consistent with the previous findings that CrGPATer could be detected in the *C. reinhardtii* lipid droplet proteome (Nguyen et al. 2011; Goold et al. 2015). Overall, these results taken together suggested that the eukaryotic Kennedy pathway constitutively contributed to the TAG biosynthesis in *C. reinhardtii.* It is noteworthy that the potential sterol:acyl-CoA acyltransferase activity of CrGPATer remains to be investigated in future studies.

Interestingly, the CrGPATer KD lines also showed increases in the TAG content (Fig. 5B). According to the RT-qPCR result, we speculated that the increased TAG accumulation was attributable to the spontaneous upregulation of the prokaryotic type CrGPATcp and CrLPAAT1 in the CrGPATer KD lines (Fig. 5C). The recent study on *CrDGAT* knockout mutants found that knockout of *DGTT1* triggered upregulation of the *CrPDAT* gene, resulting in the increase of TAG content in CrDGAT KO lines (Lee et al. 2022). This indicated that there might be compensation mechanisms governing lipid homeostasis, particularly for the TAG biosynthesis in *C. reinhardtii*. Another possible scenario was that the assimilated carbons and energy in CrGPATer KD lines were rechanneled from DNA/proteins biosynthesis to TAG biosynthesis due to reduced cell division. Different from the pattern of TAG accumulation in the CrGPATer OE lines, the TAG content of CrGPATer KD lines increased mainly on Days 2 and 3 under N-depletion (Fig. 5B), indicating that the prokaryotic pathway may play the major role in TAG biosynthesis during the late stage of N-depletion. It has been reported that, under the sustained nitrogen-depleted conditions, the galactolipids were partially degraded and transformed into TAG through the mediation of plastid galactoglycerolipid lipase (PGD1) in *C. reinhardtii*. The fatty acids hydrolyzed from galactolipids could be assembled to form TAG via the prokaryotic pathway. Such a lipid conversion process was thought to alleviate a detrimental overreduction of the photosynthetic electron transport chain (Li et al. 2012; Du et al. 2018). In this study, the chlorophyll fluorescence parameters were measured for the CrGPATer KD lines grown under the nitrogen-depleted conditions. The results showed that *F*_v_/*F*_m_ (the maximum quantum yield of photosystem II) of the CrGPATer KD lines was 0.52, 85% of the control group. At the same time, for the samples on Day 3 under nitrogen-depleted condition (N3), CrGPATer KD lines also showed decreases in the quantum yield of PSII under the light conditions in the range of 35 to 219 *μ*mol photons m^−2^ s^−1^, which indicated that the photosynthesis was somehow impaired in the CrGPATer KD lines (Supplemental Fig. S2). Thus, the prokaryotic Kennedy pathway is likely indispensable for protecting microalgae from photo-oxidative stresses, which can explain the spontaneous upregulation of the genes encoding the acyltransferases in the prokaryotic pathway in the CrGPATer KD lines.

### Eukaryotic Kennedy pathway is involved in the galactolipids synthesis in *C. reinhardtii*

The lipidome data indicated that all galactolipids composition was 18C/16C (*sn*-1/*sn*-2) in *C. reinhardtii* (Li-Beisson et al. 2015). Based on the studies of galactolipids biosynthesis in land plants, *C. reinhardtii* was considered to only use the chloroplast-derived PA to synthesize MGDG and DGDG (Kalisch et al. 2016; LaBrant et al. 2018; Hoelzl and Doermann 2019). However, a recent study indicated that *C. reinhardtii* ER-derived CrLPAAT2 also prefers C16 fatty acids as the substrate (Kim et al. 2018), so the subcellular localization of galactolipids in *C. reinhadtii* remained to be investigated.

The results of this study showed that the genetic manipulation of CrGPATer altered the content of galactolipids directly. OE of CrGPATer promoted the accumulation of MGDG and DGDG (Fig. 6, A and B), while KD of it could reduce the contents of galactolipids (Fig. 6, C and D). Moreover, it was also found that the KD of CrGPATcp in the prokaryotic pathway had no significant effect on the intracellular galactolipids content (Fig. 6, E and F). Similar results have been observed in *A. thaliana ats1* mutant (insertion mutagenesis of prokaryotic *GPAT*, *ATS1*). In *ats1*, the eukaryotic pathway could provide all kinds of lipids required for metabolic processes under normal conditions (Kunst et al. 1988; Obata et al. 2021). It indicated that the prokaryotic pathway was not the main producer of galactolipids synthesis or there might be other pathways responsible for supplying the lipid precursors (i.e. through eukaryotic pathway) required for galactolipids synthesis when the PA derived from the prokaryotic pathway was reduced.

On the other hand, in plants, such as *A. thaliana*, trigalactosyldiacylglycerols (TGDs) protein complex localized on the chloroplast envelope membrane is responsible for transporting the PA produced at the eukaryotic Kennedy pathway into chloroplast for the synthesis of galactolipids (Fan et al. 2015). And this lipid transportation probably also exists in *C. reinhardtii*. The genome of *C. reinhardtii* encodes *CrTGD1∼3* which is homologous with *A. thaliana* TGD1∼3, respectively, and CrTGD2 has been proved to specifically bind with PA in vitro (Warakanont et al. 2015). However, the transport mechanism via TGDs depends on the multi-subunit protein complex (i.e. AtTGD1∼5) (Yu et al. 2021), and it remains to be verified by experiments that the putative CrTGD1 and 3 play a similar role in PA transporting in *C. reinhardtii.* In this study, *C. reinhardtii* cells were fed with deuterated PA (d31-PA) with the aim to investigate whether the extraplastidial PA could be imported into the chloroplast for galactolipids synthesis. Although the stereochemical structure of this PA molecule is most likely different from the *C. reinhardtii* endogenous PA (*sn*-1/*sn*-2: 18C/16C), our results showed that the labeled fatty acid chain (d31-16:0) was integrated into the galactolipid molecules (Fig. 7, B and C), but at extremely low efficiency. This can explain why a trace amount of labeled DGDG (i.e. 150 pmol·g^−1^) was produced in the feeding experiment (Fig. 7D). In chloroplasts, DAG dephosphorylated from PA is catalyzed by MGD to synthesize MGDG, and MGDG is further synthesized into DGDG through DGD (Li-Beisson et al. 2019). After feeding d31-PA for 3 h, the content of the labeled MGDG quickly reached the maximum value, which indicated that the chloroplast rapidly synthesized MGDG by utilizing the fed d31-PA. After reaching the plateau, its content gradually decreased, indicating that the labeled MGDGs were then transformed into DGDG. During the first 3 h after feeding, the content of labeled DGDG gradually increased, indicating that the exogenous-labeled MGDGs were continuously used to synthesize the DGDG. From 3 to 6 h, owing to the synthesis of precursor material reaching saturation, the synthesis of DGDG slowed down. After 12 h of feeding, the labeled galactolipids content gradually leveled off. In conclusion, it was suggested by the alterations of the labeled MGDG and DGDG contents that the d31-PA was utilized as the precursor to synthesize MGDG and then to DGDG. In summary, these results indicated that the eukaryotic Kennedy pathway could provide lipid precursors for galactolipids synthesis in *C. reinardtii*.

### CrGPATer is a promising target for enhancing the production of the functional lipid OPO

OPO is one of the major TAG species in human milk fat and has been used as additives in infant formula (Wei et al. 2019). In the conventional OPO production technical route, the immobilized recombinant *sn*-1/3-regioselective lipase is used to convert plant oils, in which the *sn*-2 position of TAG glycerol backbone is esterified with oleic acid (C18:1) and palmitic acid (C16:0) to the outer (*sn*-1/3) position, into OPO with distinctive stereochemistry. Such a process is complex and costly, and is not environment-friendly due to producing organic solvent waste. (Zou et al. 2016). Many efforts have been made to produce the TAG molecules with the C16:0 at the *sn*-2 position by using engineered microorganisms such as *Rhodococcus opacus* (Zhang et al. 2020) or rewiring the metabolic pathways of oil crops to produce OPO (van Erp et al. 2019, 2021). However, none of these technologies have been commercialized and more economic-viable and environment-friendly technologies are desirable.

This study is a proof of concept that the microalga *C. reinhardtii* is capable of producing OPO. Under the mixotrophic conditions, the wild type (cc400) contained ca. 1% OPO per dry weight, which accounted for 10% of the total TAG on Day 3 under N-depleted conditions (Fig. 8, B and C). Compared with other cell factories, *C. reinhardtii* offers many advantages, including its potential for highly efficient carbon fixation and high cell-density cultivation under the heterotrophic conditions (Zhang et al. 2019). In addition, *C. reinhardtii* is a well-established synthetic biology chassis, which can be further engineered for biomanufacturing high-value products including OPO.

In this study, it is demonstrated that CrGPATer is a potential genetic engineering target for improving OPO content and yield. In the CrGPATer OE lines, the OPO content is 80% higher than that of the control (Fig. 8B), and CrGPATer OE increased the percentage of OPO in total TAG, which indicated that the OPO biosynthesis was specifically enhanced in the CrGPATer OE lines. It is believed that the content of OPO in *C. reinhardtii* could be further improved through rational genetic engineering. In fact, there are several key enzymes involved in glycerolipids synthesis (like ER-located CrLPAAT2) that are promising targets for enhancing OPO production as well. On the other hand, the synthetic biology tools can be leveraged to engineer *C. reinhardtii* for OPO production, such as expressing the exogenous or designed OPO module, redesigning and optimizing carbon flow to promote OPO assembly through artificial intelligence-assisted calculation, and screening the transgenic algae by using high-throughput and noninvasive technology.

## Materials and methods

### In vitro enzyme assays

Heterologous expression of CrGPATer in yeast (*S. cerevisiae*), yeast cultivation, and protein preparation was performed as described in Supplemental materials and methods. Regular acyl-CoAs (16:0-CoA, 18:0-CoA, 18:1-CoA, 18:2-CoA, and 18:3-CoA) were purchased from Avanti Polar Lipids, Inc (USA). The substrate 16:0 DCA-CoA was synthesized using 16:0 DCA, carbonyldiimidazole, and tetrahydrofuran as described in Kawaguchi (1981). The CrGPATer enzyme assay was performed according to the method reported previously (Yang et al. 2010; Chen et al. 2014) with slight modification. In brief, 20 µg of yeast microsomal fractions were incubated with 0.5 mM [^14^C (U)] glycerol-3-phosphate (0.1 *μ*Ci) and 45 *μ*M acyl-CoA in a buffer containing 37.5 mM Tris-HCl (pH 7.5), 2 mM MgCl_2_, 4 mM NaF, 1 mM DTT, and 0.1% (w/v) BSA at 22 °C with 750 rpm agitation for 0 to 60 min. The reactions were quenched with 5 *μ*L of acetonitrile: acetic acid (4: 1, v/v). The entire reaction mixture was loaded immediately to a TLC plate (layer: 0.25 mm, SIL G-25; DC-Fertigplatten), which was subsequently developed in a solvent system of chloroform:methanol:acetic acid:water (85: 15: 10: 3.5, v/v). Radiolabeled product LPA was identified by co-migration with 16:0-LPA standard and scraped from a TLC plate and subjected to scintillation counting.

To distinguish the regiospecificity of LPA, the dephosphorylation of LPA was performed according to the method reported previously (Yang et al. 2010). Immediately after CrGPATer assay, 0.1 M borate buffer (pH 7.5, 1 mL) and 0.1 M Tris·HCl (pH 7.5, 1 mL) were added. Dephosphorylation was started by adding 1 *μ*L of *E. coli* alkaline phosphatase (0.25 unit) and continued for 30 min at 22 °C. Products were extracted by diethyl ether and loaded on borate-TLC with 16:0 *sn*-1 and *sn*-2 MAGs as standards (Yang et al. 2010). The TLC plate was developed in a solvent system containing chloroform:acetone:methanol (96:3.5:0.5, v/v).

### Algal strains, culture conditions, transformation, and transgenic lines establishment

The cell wall-defective *C. reinhardtii* strain cc400, ordered from *Chlamydomonas* Resource Center (www.chlamycollection.org, Minnesota University), were used as recipient strains for transformations with the recombinant OE plasmid of *CrGPATer* and gene silencing plasmid of *CrGPATer* and *CRGPATcp* (recombinant plasmid were performed as described in Supplemental materials and methods). Strain were grown mixotrophically in TAP medium (Gorman and Levine 1965) on a rotary shaker at 25 °C and approximately 50 *μ*mol photons · m^−2^ · s^−1^, which was measured by using a photosynthetically active radiation meter (Mastercycler proS, Eppendorf, Germany).

For nitrogen deficiency induction, the cell culture that had grown 3 d in TAP medium with an initial cell concentration of 2.5 × 10^5^ cells·mL^−1^ would be centrifuged at 1,500 × *g* for 10 min at room temperature and resuspended to the same cell density by using TAP·N-depleted (without NH_4_Cl) medium.

For transformation, 2 × 10^8^ cells of cc400 in 300 *μ*L were agitated with glass beads (Kindle 1990) in the presence of 1 *μ*g plasmid linearized with *ScaI*, and incubated on TAP agar plates containing 10 *μ*g·mL^−1^ paromomycin (Sangon Biotech, China). Normally, single colonies would grow in 7 d after transformation and its gDNA extraction by using 5% (w/v) Chelex-100 reagent (Cao et al. 2009). For positive colony screening, primers of P4-aphVIII-F/P4-3′UTR-R and P3-F/P3-R (Supplemental Table S1) were used for OE and KD transformants screening, respectively.

For transgenic line establishment, whether the inserted recombinant fragment is complete would be verified by PCR at first, and then the target gene expression of these transformants was measured by RT-qPCR and Western blotting (see Supplemental materials and methods). Finally, those mutants that exhibited stable target gene expression both at transcription and translation levels were identified as OE or KD transformants.

### Lipid extraction and component analysis

Total lipids extraction and GCMS measurement were performed according to the method described by Wu et al. (2019). For TAG determination, firstly, it was separated from total lipid via TLC with ether:diethyl ether:acetic acid (80:20:1, v/v); secondly, after stained with iodine, areas corresponding to TAG standards (Sigma-Aldrich, USA) of each sample on the TLC plate were scraped down and the remained lipid on it was solubilized in chloroform:methanol (1:1, v/v); and finally, the samples would be processed following above-mentioned method.

UPLC-MS/MS was the analytical tool for qualitative and quantitative analysis of *C. reinhardtii* polar lipids by using the protocol described previously (Zang et al. 2019).

### D31-PA feeding

About 10 mg 16:0-d31-18:1 PA (Avanti, USA) was resuspended in 5 ml chloroform to 2.747 *μ*mol·mL^−1^ (same as 2 mg·L^−1^) as stock solution. Before feeding, 165 and 330 *μ*L of d31-PA stock solution were added into 2 individual 50 mL triangular flasks, respectively. After drying with nitrogen gas flow, 15 mL of mid-logarithmic phase cc400 cell culture was added to each vial and the group without d31 PA was set as the control group. *Chlamydomonas reinhardtii* cells were cultured in a rotary shaker at 25 °C and approximately 50 *μ*mol photons·m^−2^ · s^−1^ for 24, and 3 mL of the cell culture was sampled at each time point, marked as 0, 3, 6, 12, and 24 h. After freeze-dried, the total lipid of each sample was extracted according to the above-mentioned method and resuspended in chloroform:methanol (1:1, v/v). Polar lipid separation processed by TLC with chloroform:methanol:acetic acid:water (85:20:10:4) (Lu et al. 2007). After being stained with iodine, the area corresponding to galactolipids standards (Sigma-Aldrich, USA) was scraped down and was analyzed qualitatively and quantitatively by the above-mentioned GCMS method.

### Accession numbers

Sequence data from this article can be found in the GenBank/EMBL data libraries under the following accession numbers: *Crα-tubulin* (M11447), *CrGPATer* (XM001696416), and *CrGPATcp* (XM043060202).

## Supplementary Material

kiad091_Supplementary_Data

## Supplemental data

The following materials are available in the online version of this article.

**Supplemental Figure S1.** Knockdown of CrGPATer in *C. reinhardtii* strain cc400.

**Supplemental Figure S2.** Chlorophyll a fluorescence parameters of the CrGPATer KD lines and control group.

**Supplemental Table S1.** List of primers used in this study.
