# Supplementary material for: Microalgal glycerol-3-phosphate acyltransferase role in galactolipids and high-value storage lipid biosynthesis
Source: Plant Physiol. 2023 Feb 21;192(1):426–41. doi: 10.1093/plphys/kiad091 (PMC10152646; doi:10.1093/plphys/kiad091)
Supplement: kiad091_Supplementary_Data [file kiad091_supplementary_data.pdf]

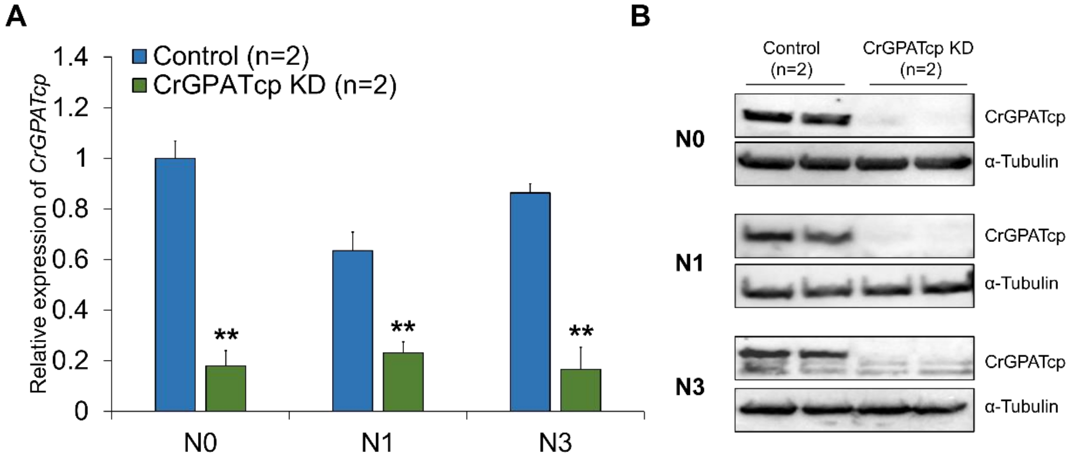

Supplemental Figure S1. Knockdown of CrGPATcp in *C.reinhardtii* strain cc400. A and B, Relative mRNA expression level of *CrGPATcp* (A) and its protein abundance (B) among control group and CrGPATcp KD lines during nitrogen-replete (N0) and nitrogen-depleted (N1, N3) conditions. qPCR data normalized to target gene expression in control group at N0. α-Tubulin were as reference both in RT-qPCR and WB assay. Control (n=2) group contains wild type and one empty vector transformant and CrGPATcp KD (n=2) group contains two individual knockdown transformants. Every kinds of strain had two biological parallels in culture cycle. Data are means of replicates with SD. Asterisks indicate statistically significant differences from control group based on Student's t test (*: $P < 0.05$, **: $P < 0.01$).

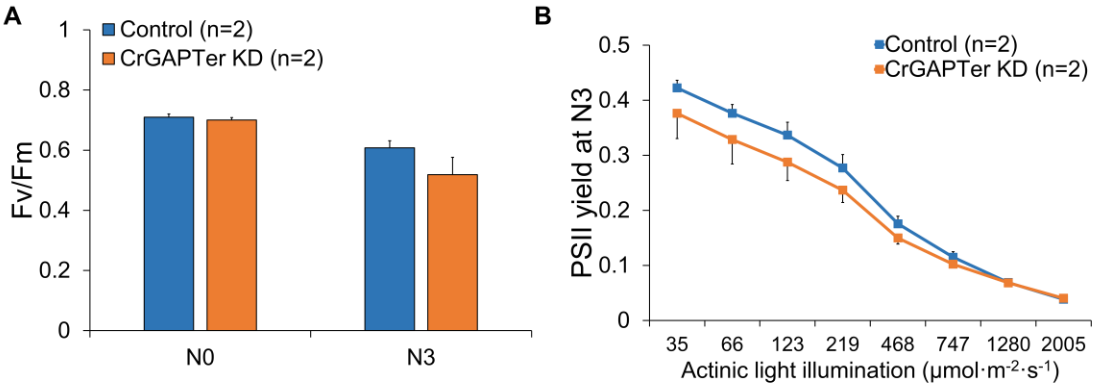

Supplemental Figure S2. Chlorophyll a fluorescence parameters of the CrGPATer knockdown (KD) lines and control group. A, The maximum quantum efficiency of PSII photochemistry, Fv/Fm of CrGPATer KD lines and control group both under the nitrogen-replete (N0) and nitrogen-depleted (N3) conditions; B, The actual quantum efficiency of PSII photochemistry, Y(II) of N3 samples, based on rapid light curve measurement. Control (n=2) contain wild type cc400 and one empty vector transformant. CrGPATer KD (n=2) contain two individual knockdown transformants. Every kind of strain had two biological parallels in culture cycle. Data are means of replicates with SD.

Supplemental materials and methods

Heterologous expression of CrGPATer in yeast, yeast cultivation, and protein preparation

The resulting PCR product of *CrGPATer* was inserted downstream of the GAL1 promoter in the pYES2.1 TOPO®TA yeast expression vector (Thermo Fisher Scientific, USA) according to the manufacturer's instructions and was confirmed by sequencing. *S. cerevisiae Δgat1* (*BY4742, Matα, his3Δ1, leu2Δ0, lys2Δ0, ura3Δ0, YKR067w::kanMX4*) (Zheng et al., 2003) was transformed with both the experimental construct as well as the empty vector control, respectively, according to the manual of the pYES2.1: V5-His-TOPO TA expression kit (Invitrogen, USA). Yeast cells were harvested in 18 hours by centrifugation at 1500 g for 5 min. The resulting cell pellets were washed with ice-cold 20 mM Tris·HCl (pH 7.9) once and resuspended in 1 mL of breaking buffer [20 mM Tris·HCl (pH 7.9), 10 mM $MgCl_2$, 1 mM EDTA, 5% (v/v) glycerol, 1 mM DTT, 0.3 M ammonium sulfate] (Yang et al., 2010).

The cells were then broken with a Mini-Beadbeater (BioSpec Products USA) and the crude homogenates were centrifuged at 12,000 g at 4°C for 10 min to remove the cell debris. The supernatant was further centrifuged at 100,000 g at 4°C for 70 min to separate microsomal and cytosolic fractions. The microsomal pellets were washed and resuspended in 50 mM Tris·HCl (pH 7.9) buffer containing 20% (v/v) glycerol and 1mM DTT, flash frozen with liquid nitrogen, and stored at −80°C for CrGPATer assay.

Bioinformatics analysis

The software SignalP-5.0 (Armenteros et al., 2019), TargetP-2.0 (Armenteros et al., 2019) and ProtComp-9.0 (http://linux1.softberry.com/) were used to predict the localization of CrGPATer.

Overexpression and knock-down recombinant plasmid construction

The putative fragment, *ble*, in target gene OE plasmid, pChlamy4 (Invitrogen, USA) was exchanged by *aphVIII* which shows paromomycin resistance. To integrate the *CrGPATer* into the modified pChlamy4 vector, firstly, the full-length cDNA of

CrGPATer was amplified from the homemade cDNA library by using the primer sets *CrGPATer*-F and *CrGPATer*-R (Supplemental Table S1) and the sequence of lowercase letters were homologous arms of empty vector. Secondly, empty vector was linearized by PCR via P4-F and P4-R (Supplemental Table S1). Lastly, purified empty vector mixed with *CrGPATer* CDS fragment maintained homologous arms sequence of vector and achieved homologous recombination followed by *pEASY*®-Basic Seamless Cloning and Assembly Kit protocols (Transgene, China). All of above mentioned PCR reactions were carried out by using Phanta Master Mix high-fidelity DNA polymerase (Vazyme, China) according to the manufacturer's instructions.

Plasmid for *C. reinhardtii* gene silencing, pChlamiRNA3int ordered from *Chlamydomonas* Resource Center (www.chamycollection.org, Minnesota University). And in order to screen the knock down transformants rapidly, followed by Hu's method (Hu et al., 2014), the luciferase gene from the marine copepod *Gaussia princeps* (G-Luc) was amplified from the plasmid pHK226 (kindly provided by Kaiyao Huang's Lab) using primers PsaD-Luc F and RBSC2-Luc R (Supplemental Table S1), which included homologous flanking sequence of PsaD promoter 3' ends and RBCS2 intron1 5' end. Similarly, the RBCS2 fragment come from pChlamiRNA3int was amplified by using primers Luc-RBCS2 F and MIR1157-RBCS2 R (Supplemental Table S1), which are flanked by an homologous sequence at G-Luc 3' terminus and cre-MIR1157 5' terminus. The linearized pChlamiRNA3int plasmid by *NdeI* (Invitrogen,USA) mixed with above two remain homologous flaking sequence and followed by *pEASY*®-Basic Seamless Cloning and Assembly Kit protocols (Transgene, China), yielding new plasmid, pChlamiRNA3int: Luc. It had been sequenced by primers of PsaD F2 and MIR1157 R (Supplemental Table S1). The amiRNA targeting the *CrGPATer* and *CrGPATcp* in *C. reinhardtii* was designed using WMD3 software (wmd3.weigelworld.org) (Ossowski et al., 2008). The output oligonucleotide was a 96 bp amiRNA precursor sequence (Supplemental Table S1), remaining *SpeI* sticky ends and these DNA sequence synthesized by GenScript Company (China). Next, the precursor of amiRNA was inserted in linearized pChlamiRNA3int: Luc with *SpeI*, and

the correct orientation of the insertion was selected by sequencing result derived by primers of PsaD F2 and MIR1157 R (Supplemental Table S1).

Transmission electron microscopy

C. reinhardtii cells were harvested at 1,500 g for 10 min and fixed overnight at 4°C with a PBS buffer (pH 7.4) containing 2% (v/v) glutaraldehyde. After rinsing three times with PBS, cell samples were post-fixed with 1% (w/v) osmium tetroxide in PBS for 2 hours at room temperature. After post-fixation, the cells were dehydrated as Wayama's research described (Wayama et al., 2013). Then the cell samples were embedded and polymerized in Spurr's epoxy resin at 60°C for 48 hours. A 65 nm thin section were cut using a Leica Ultracut-R microtome and stained with 2% (v/v) uranyl acetate and Sato's lead citrate (Hanaichi et al., 1986) and examined with a Philips CM12 transmission electron microscope.

Organelle preparation

Cells grown in 500 mL TAP for 4 days and TAP-N medium for 6, 12, 24, and 48 hours were centrifuged at 1000 g for 10 min. Cells were washed in ice-cold PBS buffer and resuspended in 5 mL of isolation buffer (10 mM Tris·HCl (pH 7.5), 1 mM EDTA, 0.5 M sucrose) with 1×cocktail protease inhibitors (Sigma-Aldrich, USA). Cells were disrupted with the aid of a homogenizer, using 30 strokes on ice. The homogenates were centrifuged at 800 g for 10 min to remove unbroken cells, large debris, nuclei and intact chloroplasts. The resultant supernatant was subjected to sequential centrifugation at 3000 g for 15 min in a Beckman centrifuge J26 with a JS-5.3 rotor, and then 6000 g for 30 min (mitochondrial fraction), 100,000 g for 60 min (microsome fraction) in a Beckman L-90K centrifuge with a SW 32i rotor. Chloroplast thylakoid membranes were isolated as Gu's method described (Gu et al., 2021). Lipid bodies isolation followed by Liu's method (Liu et al., 2016).

Membrane fractions were solubilized in an extraction buffer (60mM DTT, 60mM Na_2CO_3, 2% (w/v) SDS and 12% (w/v) sucrose) by vortex for 20 min at 4°C. Protein concentration was determined using the BCA protein assay, as directed by the manufacturer (Thermo Scientific, USA).

Expression analysis by RT-qPCR

1.5 mL cell culture were harvested by centrifugation at 1,500 g for 5 min and washed twice with the 1×PBS buffer. The cell pellets were stored at -80°C prior to use. Total RNA of each sample was isolated by TransZol up Plus RNA Kit (Transgene, China). The synthesis of single stranded cDNA was carried out using TransScript All-in-One First-Strand cDNA Synthesis SuperMix for qPCR (TransGen Biotech, China). The reverse transcription quantitative real-time PCR (RT-qPCR) was performed by TransStart® Tip Green qPCR SuperMix (TransGen Biotech, China) on a LightCycler 96 (Roche, German). *C. reinhardtii α tubulin1* (Cre03.g190950) was used as an internal reference gene. Primers used in RT-qPCR analyses for relative expression of genes are shown in Supplemental Table 1. The relative expression levels were calculated using the $2^{-\Delta\Delta Ct}$ method.

Western blotting analysis

Sample preparation for SDS-PAGE were processed through Li's method (Li et al., 2020) and whole protein were separated on 12% (w/v) SDS-PAGE, and transferred to nitrocellulose filter (NC) membrane at constant 25 V, for 20 min by using semi-dry transfer system (Bio-Rad Trans-Blot Turbo Transfer System, USA). The membranes were blocked with 10 ml of 5% (w/v) nonfat milk.

For immunofluorescence detection of Bip, Aox, D1, CrGPATer, CrGPATcp and α-tubulin, the membrane was incubated overnight with rabbit primary antibody (diluted at 1: 2000 with TBST containing 2% (w/v) nonfat milk), after washed by TBST buffer three times, membrane incubated with the secondary antibody which were anti-rabbit IgG conjugated with HRP diluted by 1: 1000. Antigen-antibody complexes were visualized using an enhanced chemiluminescence detection kit (Bio-Rad Clarity Western ECL Substrate, USA).

The subcellular compartment marker antibodies against Bip, Aox and D1were ordered from Agrisera (Sweden). The tubulin was used as internal reference, which was detected by using the tubulin alpha chain antibody (Agrisera, Sweden). The CrGPATer

and CrGPATcp polyclonal antibody was generated by using heterologous expressed recombinant protein in *E.coli* as antigen (ABclonal, China).

Data processing

Two-tailed Student's t-test was used to compare the differences between control groups (wide type and one empty vector transformant) and experimental groups (target gene transformants). If the test gives *P* value ≤0.05, the differences between two samples were interpreted as being significant.

References

Armenteros JJA, Salvatore M, Emanuelsson O, Winther O, von Heijne G, Elofsson A, Nielsen H (2019) Detecting sequence signals in targeting peptides using deep learning. Life Sci Alliance **2: e201900429**

Armenteros JJA, Tsirigos KD, Sonderby CK, Petersen TN, Winther O, Brunak S, von Heijne G, Nielsen H (2019) SignalP 5.0 improves signal peptide predictions using deep neural networks. Nat Biotechnol **37:** 420-423

Gu X, Cao L, Wu X, Li Y, Hu Q, Han D (2021) A Lipid Bodies-Associated Galactosyl Hydrolase Is Involved in Triacylglycerol Biosynthesis and Galactolipid Turnover in the Unicellular Green Alga *Chlamydomonas reinhardtii*. Plants-Basel **10:** 675

Hanaichi T, Sato T, Iwamoto T, Malavasiyamashiro J, Hoshino M, Mizuno N (1986) A stable lead by modification of sato method. Journal of Electron Microscopy **35:** 304-306

Hu J, Deng X, Shao N, Wang G, Huang K (2014) Rapid construction and screening of artificial microRNA systems in *Chlamydomonas reinhardtii*. Plant J **79:** 1052-1064

Liu J, Han D, Yoon K, Hu Q, Li Y (2016) Characterization of type 2 diacylglycerol acyltransferases in *Chlamydomonas reinhardtii* reveals their distinct substrate specificities and functions in triacylglycerol biosynthesis. Plant J **86:** 3-19

Li Z, Cao L, Zhao L, Yu L, Chen Y, Yoon K-s, Hu Q, Han D (2020) Identification and Biotechnical Potential of a Gcn5-Related N-Acetyltransferase Gene in Enhancing Microalgal Biomass and Starch Production. Front Plant Sci **11:** 544827

Ossowski S, Schwab R, Weigel D (2008) Gene silencing in plants using artificial microRNAs and other small RNAs. Plant J **53:** 674-690

Wayama M, Ota S, Matsuura H, Nango N, Hirata A, Kawano S (2013) Three-dimensional ultrastructural study of oil and astaxanthin accumulation during encystment in the green alga *Haematococcus pluvialis*. PLoS One **8:** e53618

Zheng ZF, Xia Q, Dauk M, Shen WY, Selvaraj G, Zou JT (2003) Arabidopsis AtGPAT1, a member of the membrane-bound glycerol-3-phosphate acyltransferase gene family, is essential for tapetum differentiation and male fertility. Plant Cell **15:** 1872-1887

Supplemental Table S1. List of primers used in this study

Name	Sequence (5'-3')	Purpose
CrGPATer-F	gcaacccgggccccgaattcATGGTAGAAACATCTCGTGCTC	CrGPATer CDS amplification from cDNA library
CrGPATer-R	ggatgggcttgccctctagacaCTGCGGGTGCACCTG	
P4-F	TGTCTAGAGGGCAAGCCCAT	pChlamy4 palsmid Linearization
P4-R	GAATTCGGGGCCCGGGTTGC	
P4-aphVIII-F	CGACTTGGAGGATCTGGACG	Sequencing of pChlamy4 recombinant palsmid and overexpression transformants
P4-3'UTR-R	AAGCTACCGCTTCAGCACTT	
amiRNA 1	actagtTGGTGTCGATCCCCTGGACAAtctcgctgatcggcaccatgggggtggtggtgatcagcgctaTTGTTTAGGGGATCGACACCAgctagt	amiRNA for *CrGPATer*
amiRNA 2	actagtCCGCGTGGGATTACTTGTATAtctcgctgatcggcaccatgggggtggtggtgatcagcgctaTATAGAAGTAATCCCACGCGGgctagt	amiRNA for *CrGPATcp*
PsaD-Luc F	ACTGCTACTCACAACAAGCCCAT	Amplification of Gaussia princeps lucifearse homologous recombinant fragments
RBSC2-Luc R	ACTTGTTCATATGTTACGTATCGTC	
Luc-RBCS2 F	GACGATACGTAACATATGAACAAGT	Amplification of RBS2 intron 1 homologous recombinant fragments
MIR1157-RBCS2 R	CCAAAAACACCGACCCAACACCCATATGACATCCTGCAAATG	
PsaD F2	TCACCAATCGTCACACGAGC	Sequencing of pChlamiRNA3int recombinant palsmid
MIR1157 R	CGGTCTCCAAGGCTAATCCG	
P3-F	GGTGTTGGGTCGGTGTTTTT	Knock-down transformants screening
P3-R	CGGTCTCCAAGGCTAATCCG	
α-tubulin F1	CTCGCTTCGCTTTGACGGTG	qPCR for α-tubulin (Cre03.g190950)
α-tubulin R1	CGTGGTACGCCTTCTCGGC	
CrGPATer qPF2	GAAGAACCCGGGGCTTATCG	qPCR for *CrGPATer* (Cre06.g273250)
CrGPATer qPR2	GCCACCTTCTTCCAGCCG	
CrGPATcp qF3	AGACCGTGGACAAGTCCATTGG	qPCR for *CrGPATcp* (Cre02.g143000)
CrGPATcp qR3	CGCCATCCAGGGCTGTGTGAAC	
CrLPAAT1 qF1	ACATCTACTCGCTGTTCCACCTG	qPCR for *CrLPAAT1* (Cre09.g398289)
CrLPAAT1 qF1	CGGTCCACACGGTTTATCATCAC	
CrLPAAT2 qF1	CGGCACCATTGCGGATAAAG	qPCR for *CrLPAAT2* (Cre17.g738350)
CrLPAAT2 qR1	AGTCACGACAATCTGCACCG	
CrMGD1 qF1	CGTACGGGGACAAGTACGAG	qPCR for *CrMGD1* (Cre13.g585301)
CrMGD1 qR1	GGGGTTGGTGAACTGGTAGG	
CrDGD1 qF1	TGGATGTGCCGCATCTACTG	qPCR for *CrDGD1* (Cre13.g583600)
CrDGD1 qR1	CACAGCACCTTGCCCAGGAA	
CrTGD2 qF1	AACGGACACATACAAGGCCG	qPCR for *CrTGD2* (Cre16.g694400)
CrTGD2 qR1	TCTCCATGAGCTGTGTTGCG	

Supplemental Table S1. List of primers used in this study (continued)

Name	Sequence (5'-3')	Purpose
CrDGTT1 qF	CTCTGCTCATCGGCACATTG	qPCR for *CrDGTT1* (Cre12.g557750)
CrDGTT1 qR	ATATGCCACTTGCGGAAGGT	
CrDGTT2 qF	CACCGACAAATGTGCGAATT	qPCR for *CrDGTT2* (Cre02.g121200)
CrDGTT2 qR	CACATGCATCCAGCCACAGT	
CrDGTT3 qF	ACCTCGCACTTGACCCTGAA	qPCR for *CrDGTT3* (Cre06.g299050)
CrDGTT3 qR	TCATGAAGCCTACATAAATCGACATC	
CrPDAT1 qF1	GATGCCTCATCGGGTTGGAT	qPCR for *CrPDAT1* (Cre02.g106400)
CrPDAT1 qR1	TCCCGGGTCACCACTCTCAT	
CrGGGT qF2	ACTGGTGGGGCATCAACTACTAC	qPCR for *CrGGGT* (Cre03.g171050)
CrGGGT qR2	GTCTCAGTGATGTACATGGGAATG	
